# Tendon Biomimetic Electrospun PLGA Fleeces Induce an Early Epithelial-Mesenchymal Transition and Tenogenic Differentiation on Amniotic Epithelial Stem Cells

**DOI:** 10.3390/cells9020303

**Published:** 2020-01-27

**Authors:** Valentina Russo, Mohammad El Khatib, Lisa di Marcantonio, Massimo Ancora, Ralf Wyrwa, Annunziata Mauro, Torsten Walter, Jürgen Weisser, Maria Rita Citeroni, Francesco Lazzaro, Marta Di Federico, Paolo Berardinelli, Cesare Cammà, Matthias Schnabelrauch, Barbara Barboni

**Affiliations:** 1 Unit of Basic and Applied Biosciences, Faculty of Bioscience and Agro-Food and Environmental Technology, University of Teramo, 64100 Teramo, Italy; vrusso@unite.it (V.R.); amauro@unite.it (A.M.); mrciteroni@unite.it (M.R.C.); mdifederico@unite.it (M.D.F.); pberardinelli@unite.it (P.B.); bbarboni@unite.it (B.B.); 2 Laboratory of Bacteriology, Istituto Zooprofilattico Sperimentale dell’Abruzzo e del Molise “Giuseppe Caporale”, 64100 Teramo, Italy; l.dimarcantonio@izs.it; 3 Laboratory of Molecular Biology and Genomic, Istituto Zooprofilattico Sperimentale dell’Abruzzo e del Molise “Giuseppe Caporale, 64100 Teramo, Italy; m.ancora@izs.it (M.A.); c.camma@izs.it (C.C.); 4 Department of Biomaterials, INNOVENT e. V, J-07749 Jena, Germany; rw1@innovent-jena.de (R.W.); tw@innovent-jena.de (T.W.); jw1@innovent-jena.de (J.W.); ms@innovent-jena.de (M.S.); 5 Research & Development Department, Assut Europe S.p.A., Magliano dei Marsi, 67062 L’Aquila, Italy; francesco.lazzaro@assuteurope.com

**Keywords:** aligned fibers, amniotic epithelial stem cells, biomimetic scaffold, electrospinning, epithelial-mesenchymal transition, PLGA, tendon tissue engineering, tenogenic differentiation

## Abstract

*Background.* The design of tendon biomimetic electrospun fleece with Amniotic Epithelial Stem Cells (AECs) that have shown a high tenogenic attitude may represent an alternative strategy to overcome the unsatisfactory results of conventional treatments in tendon regeneration. *Methods.* In this study, we evaluated AEC-engineered electrospun poly(lactide-co-glycolide) (PLGA) fleeces with highly aligned fibers (ha-PLGA) that mimic tendon extracellular matrix, their biocompatibility, and differentiation towards the tenogenic lineage. PLGA fleeces with randomly distributed fibers (rd-PLGA) were generated as control. *Results.* Optimal cell infiltration and biocompatibility with both PLGA fleeces were shown. However, only ha-PLGA fleeces committed AECs towards an Epithelial-Mesenchymal Transition (EMT) after 48 h culture, inducing their cellular elongation along the fibers’ axis and the upregulation of mesenchymal markers. AECs further differentiated towards tenogenic lineage as confirmed by the up-regulation of tendon-related genes and Collagen Type 1 (COL1) protein expression that, after 28 days culture, appeared extracellularly distributed along the direction of ha-PLGA fibers. Moreover, long-term co-cultures of AEC-ha-PLGA bio-hybrids with fetal tendon explants significantly accelerated of half time AEC tenogenic differentiation compared to ha-PLGA fleeces cultured only with AECs. *Conclusions.* The fabricated tendon biomimetic ha-PLGA fleeces induce AEC tenogenesis through an early EMT, providing a potential tendon substitute for tendon engineering research.

## 1. Introduction

In human and animal (i.e., equine, canine) patients, tendinopathies have a high incidence and are still a persistent orthopedic challenge to be solved due the fact of their high incidence of recurrences as well as their low healing ability. Annually there are worldwide over 30 million treatments for tendon disorders, representing a related cost of over 140 billion EUR in the U.S. and EU [1,2,3,4]. Tendinopathies have shown clinical relevance also in veterinary medicine, in particular related to racehorses, generating a strong negative economic impact estimated worldwide at 400 billion EUR [5,6]. The low cellular and hypo-vascular nature of the tendon is responsible for the failure of spontaneous tendon healing that leads to a disorganized extracellular matrix (ECM) production, which compromises the biomechanical properties of the tissue. Current tendinopathy therapeutic strategies foresee the use of conservative approaches or surgical repair using autografts, allografts, and xenografts, which have a limited success [7].

More recently, this situation has prompted researchers to find alternative solutions in tendon tissue engineering. In this field, the design of a biomimetic scaffold and the choice of the cell source are two fundamental aspects to be considered. In particular, one important aspect is the correct design of non-woven fleeces that mimic the collagen parallel structure and the biomechanics of the native ECM of the tendon [8,9]. Electrospinning has been identified as a valuable manufacturing process to generate constructs mimicking the micro and submicron-scaled fibrous structure of the native ECM of various soft tissues [10,11,12,13]. Using an advantageous electrospinning technique, this process can be also used to fabricate fleeces mimicking the highly aligned collagen fibrils of native tendons and ligaments [14,15,16,17,18]. However, the scaffolds fabricated so far have shown many constraints restricting their clinical use [19,20]. The resulting electrospun fleeces generally possess a tightly packed fibrous structure and less control in fiber alignment, limiting cell biocompatibility in terms of growth, penetration, oxygen and nutrients delivery within the construct, in addition to inadequate mechanical properties, which are necessary for tendon regeneration [21,22]. Thus, these constructs do not fulfill the requirements needed to mimic a native tendon [23,24], imposing the need for new functional tendon biomimetic fleeces.

On the other hand, in stem-cell-based therapy for tendinopathies, cell compatibility of the biopolymer is an important aspect to be considered [25]. 

The mesenchymal stem cells and fibroblasts are the most commonly used cells in tendon tissue engineering [26,27,28,29]. However, their application is hindered by tendon ossification after in vivo transplantation of mesenchymal stem cells, in addition to the limited proliferative ability of fibroblasts, both of which negatively influence tissue regeneration [29,30,31]. Only recently has success been achieved in tendon regeneration by using Amniotic Epithelial Stem Cells (AECs), which are derived from the discarded placenta. They represent an attractive stem cell source due to their availability, non-ethical concerns, non-tumorigenic potential, and in vitro and in vivo differentiation aptitude especially into the tenogenic lineage [32,33]. In a highly conserved manner, these types of extra-fetal stem cells express SSEA-3, SSEA-4, TRA-1-60, and TRA-1-81 embryonic markers and OCT4, SOX2, NANOG, and TERT pluripotent genes, which are responsible for their high plasticity [34]. AECs’ immunomodulatory properties represent an important biological characteristic [35], as they can be successfully used in allo- and xeno-transplantation preclinical settings and clinical trials [32]. The natural inclination of AECs to undergo the Epithelial-Mesenchymal Transition (EMT) process [36] justifies their capacity to differentiate in vitro towards the mesenchymal lineage, as the tenogenic one, under adequate stimuli [33]. In addition, AECs transplanted in a tendon injury model are also able to teno-differentiate in vivo [32]. Thus, these cells seem to be the most appropriate cell source for tendon tissue engineering. Another important aspect is the cultural method that is able to accelerate tendon regeneration [23,37]. Barboni et al. have demonstrated that AECs co-cultured with tendon explants enhance their tenogenic differentiation due to the release of tenogenic paracrine molecules, especially when these are derived from fetal tendon explants [33]. 

These cells represent also a favorable solution for the fabrication of bio-hybrid fleeces in tissue engineering. Recently, it has been demonstrated that ovine AECs (oAECs) are biocompatible for polymeric fleeces made from polymers as poly-ε-caprolactone (PCL), polylactide (PLA), and poly(lactide-co-glycolide) PLGA copolymers. In particular, they are highly biocompatible for PLGA fleeces with encouraging results in terms of cell colonization, distribution, and proliferation within the construct [38]. Contextually, PLGA, when electrospun, becomes a fibrous device. It can be considered one of the common biodegradable synthetic polymers used for skeletal-muscle tissue engineering, especially for tendons [15,26,39,40,41,42,43,44].

Novel PLGA electrospun fleeces with highly aligned fibers were fabricated in this study using the electrospinning technique, in order to mimic the tendon ECM and its biomechanical properties. These fleeces have been fabricated by implementing the custom-made electrospinning machine with an optimized rotating drum collector, allowing the performance of high rotational speed to reproducibly generate highly aligned fibers. It was hypothesized that the high fiber alignment regulates AEC teno-differentiation. 

To this aim, matrices were engineered with these epithelial stem cells in order to verify their biocompatibility and teno-inductivity, as well as the mechanism involved in oAEC teno-differentiation. AEC activity was investigated on aligned fibers and compared with randomly oriented electrospun fibers. Finally, a bio-engineered electrospun PLGA highly aligned fiber fleece and fetal tendon co-culture approach was used to accelerate and increase tenogenic oAEC differentiation.

## 2. Materials and Methods

### 2.1. Materials

Poly(lactide-co-glycolide) (PLGA, PLG8523) was purchased from Corbion Purac, Gorinchem, The Netherlands. The molecular weight was determined by gel permeation chromatography (GPC) in chloroform with polystyrene as external standard to be M_w_ = 258,000 g/mol. Hexafluoro-2-propanol (HFIP) was obtained from Apollo Scientific, Manchester, UK with purity of 99%. All other chemicals and solvents were of analytical grade and used as received.

### 2.2. Fabrication of PLGA Fleeces 

A commercial E-Spintronic electrospinning apparatus (Erich Huber, Gerlinden, Germany) with climate control was used for the fabrication of electrospun fleeces under optimal conditions. Briefly, a spinning solution composed of 8 wt% PLGA dissolved in HFIP was prepared for producing the electrospun fleeces. The electrospinning process was carried out at about 21.5 °C and an air humidity of about 35%. A 5 mL plastic syringe was set on the pump and connected to a 35 cm PTFE tube (Intra Special Catheters, Rehlingen-Siersburg, Germany) at the nozzle, a stainless-steel straight-end hollow needle (0.4 mm). The working parameters were set as follows: 20 cm for the distance between the needle tip and the collector, 26 kV for the voltage applied on the needle, and 1.5 mL/h for the polymer solution feeding rate. An aluminum foil, placed on the metallic cylindrical drum rotating collector, with a diameter of 12 cm (related to a circumference of about 38 cm), was used to collect the produced electrospun fibers. Rotational speed of 1000 rpm was used to produce highly aligned fiber fleeces (ha-PLGA), while a speed of 100 rpm was used to produce randomly distributed fibers (rd-PLGA) fleece as a control (CTR). Each fleece (ha-/rd-PLGA) was prepared by electrospinning 500 µL of PLGA solution.

### 2.3. Characterization of PLGA Electrospun Fleeces

The fleeces’ fiber microstructure was assessed by scanning electron microscopy (SEM) with a Supra 55VP (Carl Zeiss, Jena, Germany) field-emission scanning electron microscope. A Si wafer was used as substrate material for the fibers and Au was sputtered on the specimens, ensuring sufficient electric conductivity. A SE2-Detector was used for image capturing by applying an excitation energy of about 5 keV. The SEM micrographs were analyzed with ImageJ software (NIH image) to determine the angle distribution, divided from −45° to +45°, and to calculate the average fiber diameter size measured from 100 fibers, randomly chosen, for each fleece type (n = 3 for each type of fleece).

### 2.4. Fleece Mechanical Tests 

Mechanical testing was performed using a Texture Analyzer TA.XT2i (Stable Micro Systems, Godalming, UK) with a load cell of 5 kg. Aligned and randomly oriented micro-fiber-based sheet specimens were cut into rectangular pieces of 50 mm × 15 mm (n = 5 for each type of fleece) after being dried for 48 h at room temperature (RT). Each sample was fixed to custom-made clamps and the load was applied along the direction of the fibers on the aligned fleeces. Each fleece underwent a load to failure test at an elongation rate of 1 mm/sec under dry conditions. The width and thickness of each sample were measured in order to calculate the cross-sectional area and furthermore determine the structural properties of the fleeces, as represented by ultimate stress (MPa) and failure strain (%). 

### 2.5. Fleece Sterilization and Conditioning

Electrospun rd-PLGA and ha-PLGA fleeces were cut into rectangular pieces (15 mm × 7 mm) for all the experiments, sterilized with 70% ethanol (EtOH) in 0.9% NaCl/distilled water (diH_2_O) for a few seconds, then washed with sterile phosphate buffered saline (PBS) as previously reported [38]. The fleeces were then rehydrated with cell culture growth medium (GM) containing minimum essential medium Eagle-α modification (α-MEM) supplemented with 10% fetal bovine serum (FBS), 1% ultra-glutamine, 1% amphotericin B, 1% penicillin/streptomycin and incubated at 38 °C with 5% CO_2_ for 24 h. 

### 2.6. Fourier Transform Infrared Spectroscopy (FTIR) Analysis

The FTIR spectra of the rd-PLGA and ha-PLGA fleeces were recorded before and after sterilization, and after sterilization and conditioning with GM, as well as using an FTIR spectrometer Nicolet iS10 (Thermo Fisher Scientific S.p.A., Milan, Italy) to evaluate if the sterilization procedure affected the PLGA fleeces’ chemical composition. The average spectra were collected from 64 accumulations with a resolution of 4 cm^−1^ operating in the range of 4000–650 cm^−1^. Three different locations from three samples were analyzed. 

### 2.7. Ovine AEC Isolation and Culture and Fetal Tendon Isolation

All cells and tissues were collected from slaughtered sheep of the Appenninica breed, and there is no requirement for any ethical statement. The amniotic membranes and fetal tendons were isolated from fetuses of 25–35 cm of length, at approximately 2–3 months of pregnancy as described previously [33]. Briefly, ovine AECs (oAECs) were isolated from the epithelial layer of the amniotic membrane after enzymatic digestion (0.25% Trypsin-EDTA, Sigma Chemical, St. Louis, MO, USA) as previously described [33]. Cell suspension was collected, filtered through a 40 µm cell filter, and poured into a 50 mL falcon tube containing FCS at a final concentration of 10% to inactivate Trypsin. Each falcon tube was centrifuged, and the pelleted vital cells were counted after Trypan-Blue staining by using a hemocytometer chamber. Isolated oAEC cells at a concentration of 3 × 10^3^ cells/cm^2^ were cultured in Petri dishes of 25 mm containing growth medium (GM) composed by Minimum Essential Medium Eagle-α modification (α-MEM) supplemented with 20% of Fetal Calf Serum (FCS), 1% Ultraglutamine, 1% Penicillin/Streptomycin and incubated at 38 °C with 5% CO_2_. At 70% of confluence, the cells were dissociated by 0.05% Trypsin-EDTA (Sigma Chemical, St. Louis, MO, USA) and cultured in GM at the same concentration. Before their use, the oAECs were previously characterized by flow cytometry investigations confirming negativity for hemopoietic markers (CD14, CD58, CD31, and CD45), positivity for surface adhesion molecules (CD29, CD49f, and CD166) and stemness markers TERT, SOX2, OCT4, and NANOG, a low expression for MHC class I molecules, and the absence of MHC class II (HLA-DR) antigens, as described in our previous reports [32,33,35]. For the experimental condition, oAECs (0.05 × 10^6^) were then cultured alone or seeded on sterilized fleeces in the presence of GM in incubation at 38 °C with 5% CO_2_ for different time periods (4, 24, and 48 h, or 8, 14, and 28 days, depending on the analysis).

Calcaneal tendon explants were isolated from the fetus forefeet and deprived of the tendon sheath under sterile conditions. Fetal tendons were cut into small pieces (1 mm^3^ in size) and mechanically disaggregated under a stereomicroscope with the aid of fine watchmaker forceps in order to maximize the interface between the tissue and the medium during the co-culture technique described in paragraph 2.11.

### 2.8. Cell Survival on Fleeces

The oAECs seeded on electrospun fleeces rd-PLGA (CTR) and ha-PLGA were evaluated for their viability after 24 h and 48 h of culture (n = 3 for each type of fleece/time point). For this, oAECs were incubated for 30 min with calcein AM (4 µM) viable cell green fluorescent dye and then for 10 min with propidium iodide (12 µM) dead cell red fluorescent dye. Cell viability was observed using an Axioskop 2 Plus incident light fluorescence microscope (Carl Zeiss, Oberkochen, Germany) equipped with a CCD camera (Axiovision Cam, Carl Zeiss) with a resolution of 1300 × 1030 pixels, configured for fluorescence microscopy, and interfaced to a computer workstation, provided with an interactive and automatic image analyzer (Axiovision, Carl Zeiss). Cell viability was calculated by counting calcein-AM-positive cells/100 cells and propidium-iodide-positive cells/100 cells. Digital images were acquired using standard filters setup for Cy3, Alexa Fluor 488 or DAPI. 

### 2.9. Ovine AEC Spatial Distribution, Morphology, and Proliferation Index 

Immunohistochemistry (IHC) was carried out to investigate the oAEC-engineered fleeces after 24 h and 48 h of culture (n = 3 for each type of fleece/analysis/time point):

- Cell morphology using phalloidin staining and SEM observation;
- Cell proliferation index (PI) by evaluating Ki-67 positivity;
- Cytokeratin 8 and a-SMA expression to evaluate the EMT;
- *COL1* expression to evaluate tenogenic differentiation.

Cells were fixed in 4% paraformaldehyde/PBS (10 min) and permeabilized in 0.05% Tween 20/1% BSA/PBS for 10 min at RT. After washing with PBS, non-specific binding was blocked, incubating the seeded PLGA fleeces with oAECs at RT for 1 h followed by incubation with the primary antibodies diluted in PBS, shown in Table 1, overnight at 4 °C. Finally, cells were exposed to Cy3 or Alexa Fluor 488 conjugated secondary antibodies diluted in PBS, as shown in Table 1, at appropriate dilutions for 40 min at RT. Nuclear counterstaining was obtained with DAPI (Vectastain) in PBS used at the final dilution of 1:5000 for 15 min at RT. In all experiments, non-immune serum was used in place of the primary antisera as a negative control. All controls performed were negative.

Cell samples were analyzed using an Axioskop 2 Plus incident light fluorescence microscope (Carl Zeiss) equipped with a CCD camera (Axiovision Cam, Carl Zeiss), as described above. 

Proliferation index was calculated by counting Ki-67 positive cells/100 cells. Moreover, EMT was evaluated by calculating Cytokeratin-8 or α-SMA positive cells/100 total cells.

Changes in cell morphology on different fleeces were visualized under a Nikon Ar1 laser confocal scanning microscope (Nikon, Düsseldorf, Germany) equipped with the NIS-Element software, using a Plan Apo λ 40X oil objective (numerical aperture 1.3; zoom 1.00X; Refractive Index: 1.515; pinhole size: 12.8 µm; pixel size = 0.63 µm; 1 picture every 0.15 s). We used the different channels as follows:

Channel 1: DAPI; λ_exc_ = 404 nm; λ_em_ = 450/50 nm, at 81% of the maximum laser power.

Channel 2: TRITC; λ_exc_ = 561.5 nm; λ_em_ = 595/50 nm at 0.6% of the maximum laser power.

Ovoid and elongated cells were distinguished using the length and elliptical form factor as described previously [45]. Briefly, the ratio of length to breadth of the object (cell) was considered as the elliptical form factor. The reference values for elongated cells were length >30 and elliptical form factor >1.7 while those for ovoid cell were length <25 and elliptical form factor <1.5.

Moreover, the morphology of oAECs in terms of cell alignment on the electrospun ha-PLGA fleeces was assessed by SEM analysis. Briefly, engineered oAEC-ha-PLGA fleeces after 48 h of culture were fixed in 1% paraformaldehyde/0.5% glutaraldehyde/0.1 M cacodylate pH 7.4 at 4 °C for 1 h at 4 °C. The samples were then washed in 0.15 M cacodylate pH 7.4 at 4 °C and dried with the critical point dryer (Emitech K850, Quorum Technologies, Lewes, UK). The samples were Au coated and analyzed at ×1600 magnification using a Philips SEM 515 by applying an excitation energy of about 15.0 kV. 

In addition, cell penetration for cells cultured on both type of fleeces was assessed by acquiring XZ projections from confocal microscopy Z-stacks at a magnification of 40X, to it was applied the depth-coded MaxIP (Maximum Intensity Projection). This image analysis option applies a color gradient to the pixels with the highest intensity values of the Z-sequence.

### 2.10. DNA Extraction and Quantification

Total genomic DNA was extracted from 0.05 × 10^6^ oAECs and from the seeded fleeces (n = 3 for each type of sample/time point) at 4 h and 48 h by Maxwell 16 cell DNA purification kit, according to the manufacturer’s instructions (Promega, Madison, WI, USA). 

All samples were analyzed using a fluorescence-based DNA quantification approach that utilizes the fluorescent property of nucleic acid binding dyes. The Qubit Quantitation Platform calculates concentration based on the fluorescence of the Qubit^®^ dsDNA HS Assay (Life Technologies™, Carlsbad, California, USA), which binds to double-stranded DNA.

### 2.11. Gene Expression Profile by RT-qPCR

The reverse transcriptase quantitative real-time polymerase chain reaction method (RT-qPCR) was performed in order to compare the mRNA expression of specific genes in oAECs cultured on Petri dishes and oAEC-engineered rd-PLGA (CTR) and ha-PLGA fleeces. Total RNA was extracted from cells by using the Direct-zolTM RNA MiniPrep Plus system Kit (Zymo Research, CA, USA) following manufacturer’s guidelines. To evaluate the RNA concentration, all samples were measured using a fluorescence-based RNA quantification approach (QubitTM RNA HS Assay, Thermo Fisher Scientific Inc.). The analysis of the *Vimentin* and *Snail 1* genes was performed as described in a previous publication [36] at 24 h, 48 h of culture; the *COL1* and *TNMD* genes were analyzed according to ex novo methods 4 h, 24 h, 48 h, 8 days, 14 days and 28 days of culture (n = 3 for each type of sample/time point). Briefly, the sequences of *COL1* and *TNMD* genes were retrieved from the GenBank database (http://www.ncbi.nlm.nih.gov/Genbank/index.html) and aligned using the DNAStar software package (DNAStar Inc., Madison, WI, USA). Primers and probes were designed and verified by the Primer Express 3.0.1 software test tool (Applied Biosystems) then synthesized by Eurofins Genomics (Ebersberg, Germany). Afterwards RT-qPCR was performed by using SuperScript^®^III Platinum^®^ One-Step RT-qPCR System (Invitrogen, Carlsbad, California, USA), adjusting the manufacturer instruction to a final volume of 25 μL and carrying out with QuantStudio 7 Flex (Life Technologies™). The thermal profile consisted of a single cycle of reverse transcription at 50 °C for 15 min followed by a denaturation step at 95 °C for 2 min for reverse transcriptase inactivation and DNA polymerase activation. The amplification of cDNA was performed by 40 cycles, including denaturation at 95 °C for 15 s, and annealing at 60 °C for 30 s. The relative expression level of mRNA was calculated by the ΔCt method. For details on primers and probe sequences see Table 2.

### 2.12. Co-Culture Systems with Fetal Tendon Explants/PLGA Fleeces Engineered with oAECs 

The co-culture system was performed as described in a previous work [33], with minor modifications. In detail, trans-well chambers (pore size 0.4 mm; Costar, NY, USA) containing fetal tendon explants (two to three explants of 1 mm^3^ in size/trans-well) were inserted into the well plates. Ovine AECs seeded on either rd-PLGA or ha-PLGA fleeces were positioned in the well plates. The incubation was performed in α-MEM supplemented with 10% FCS in 5% CO_2_ and air at 38 °C for 14 and 28 days (n = 3 for each type of co-cultured fleece/time point). Half of the medium was changed every 3 days during the first week of culture and every 1–2 days from then on. 

For the evaluation of oAEC COL1 protein expression after the co-culture with fetal tendon explants, IHC was carried out as described in the relative section, whereas mRNA *COL1* and *TNMD* expressions were determined through RT-qPCR as already described above.

### 2.13. Statistical Analysis

The quantitative data were obtained by analyzing each sample in triplicate for each analysis, expressed as mean ± Standard Deviation (±SD). The results were firstly assessed for normal distribution using D’Agostino and Pearson tests. Data sets were compared using one-way ANOVA multi-comparison tests followed by Tukey post hoc tests (GraphPad Prism 6, GraphPad Software, San Diego, CA, USA). The analysis carried out for statistically assessing the mechanical parameters used the two-tailored independent t-test (GraphPad Prism 6, GraphPad Software, San Diego, CA, USA). The values were considered statistically significant for at least *p* < 0.05.

## 3. Results

### 3.1. PLGA Fleece Characterization: Morphology, Mechanical Properties and Chemical Composition

**Figure 1:**
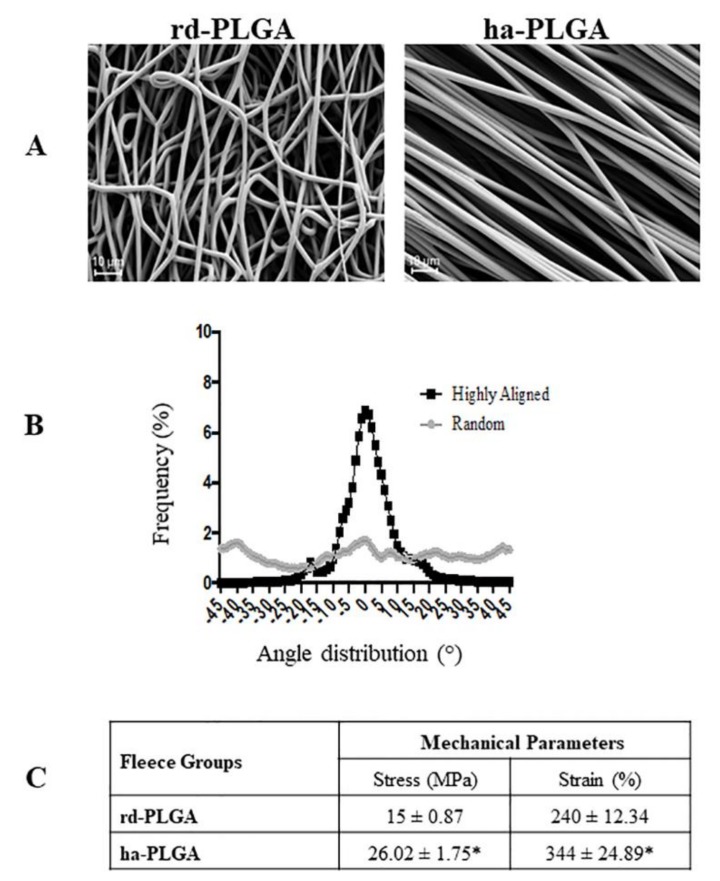
Structural and mechanical characteristics of electrospun poly(lactide-co-glycolide) (PLGA) microfibers. (**A**) SEM micrographs of the electrospun PLGA random-oriented (rd-PLGA) and highly aligned (ha-PLGA) fibers showing defect free fleeces (n = 3 for each type of fleece). Scale bars = 10 µm. (**B**) Frequency distribution of fiber orientation within PLGA fleeces with randomly distributed or highly aligned fibers. By increasing the rotational speed of the rotator collector, fibers become parallel, showing a sharp Gaussian curve where their orientation is mainly ranging within an angle from −10° to +10° differently to that of the fibers with random distribution with angles ranging from −45° to + 45; (n = 3 for each type of fleece). (**C**) Mechanical tests: stress (MPa) and strain (%) carried out on rd-PLGA and ha-PLGA fleeces (n = 5 for each type of fleece, scaffold dimension: 50 mm × 15 mm). Electrospun ha-PLGA fleeces possess higher mechanical properties compared to those for rd-PLGA. * Statistically significant values between rd-PLGA and ha-PLGA fleeces (*p* < 0.05).

PLGA fleeces, produced by electrospinning, showed defect-free cylindrical fibers in both fleece topologies (Figure 1A). The diameters of the fibers of both fibrous fleeces were comparable, in which the average fiber diameter size was 2.5 ± 0.27 µm and 2.1 ± 0.19 µm for highly aligned (ha) and randomly distributed (rd) electrospun PLGA fleeces, respectively (*p* > 0.05). Changing the rotational speed of the rotator collector led to more aligned fibrous fleeces compared to the random ones (Figure 1A). The fibers in aligned fleeces were mainly formed within an angle from −10° to +10° with respect to the tangential direction of the rotator collector (Figure 1B), hence they can be considered to be parallel, while those in random fleeces displayed an almost uniform distribution at all measured angles (Figure 1B). 

The mechanical properties of the ha-PLGA and rd-PLGA fleeces were measured in terms of ultimate stress (MPa) and failure strain (%). The mechanical properties of the aligned fleece showed much higher values than the random fleece. In particular, as shown in Figure 1C, ha-PLGA demonstrated greater stress (26 ± 1.75 MPa) and strain (344 ± 24.89%) values compared to rd-PLGA (CTR) fleece (15 ± 0.87 MPa and 240 ± 12.34%), respectively (*p* < 0.05). 

To assess if there was an effect due to the sterilization and the conditioning procedures applied on the functional groups of both types of the PLGA fleeces, FTIR technique was employed. The whole FTIR spectra demonstrated that there was no difference between rd- and ha-PLGA fleeces before and after sterilization, as well as after sterilization and conditioning with the cell culture growth medium both in the shape and in the position of the absorption peaks.

**Figure 2:**
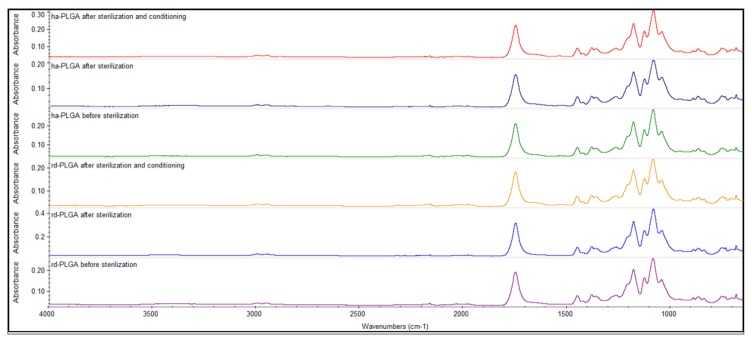
Representative FTIR spectra of rd- and ha-PLGA fleeces before and after sterilization, and after sterilization and conditioning. The spectra show that the sterilization and conditioning procedures did not alter the chemical composition of neat PLGA fleeces for both random and highly aligned fibers. Notable functional group assignments are as follows: band at 1748 cm^−1^ corresponds to ester carbonyl stretch (C=O), band at 1085 cm^−1^ corresponds to ether group stretching (C–O–C) and bands at 1452 and 1044 cm^−1^ correspond to methyl stretching (C–H) and (C–CH_3_) groups, respectively.

In detail, Figure 2 shows the main bands in the spectrum of tested PLGA fleeces. Before sterilization, a strong ester carbonyl stretch (C=O) absorption band at 1748 cm^−1^, an ether group stretching (C–O–C) at 1085 cm^−1^ and methyl stretching (C–H) and (C–CH_3_) groups at 1452 and 1044 cm^−1^, respectively, could be detected, characterizing the PLGA material. These same bands could be also observed in the spectra corresponding to the PLGA fleeces obtained after sterilization and after sterilization and conditioning in the GM without any switch or modification of the wave numbers.

### 3.2. PLGA Fleece Biocompatibility for oAECs 

Fleece biocompatibility was evaluated by seeding oAECs for 4 h and 48 h in different culture conditions (as default cultured on a Petri dish, on rd-PLGA or ha-PLGA fleeces) and assessing cell survival, distribution, morphology and proliferation. 

**Figure 3:**
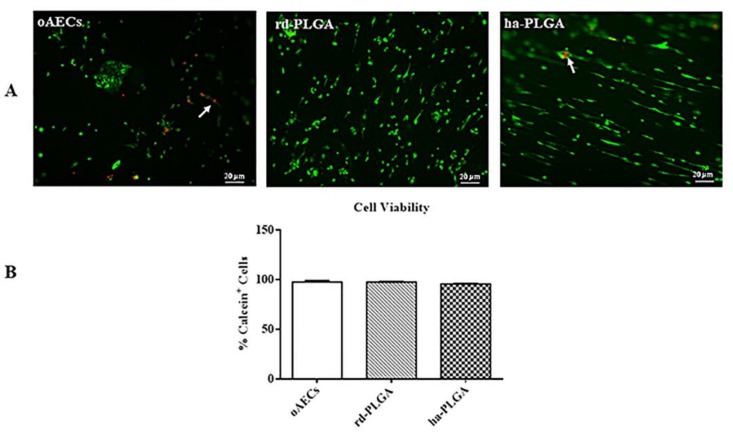
Amniotic Epithelial Stem Cell (AEC) viability on PLGA fleeces. (**A**) Representative images assessing oAEC survival with Calcein AM/propidium iodide (PI) stains (green and red fluorescence, respectively). White arrows indicate positive cells to PI. Scale bars = 20 μm (**B**) Histogram showing oAEC viability of standardly cultivated cells on a Petri dish (oAECs) and on rd-PLGA or ha-PLGA fleeces after 48 h culture. No statistical difference was evident among the three groups (*p* > 0.05); (n = 3 for each type of fleece/time point, scaffold size: 15 mm × 7 mm).

Cell viability was studied by using Calcein AM (alive cell marker, green fluorescence) or Propidium Iodide stain (dead cell marker, nuclear red fluorescence). As shown in Figure 3A, after 48 h of culture period, green fluorescent cells in live/dead staining showed that both PLGA fleeces were not cytotoxic, indicating high cell survival and integration capacity within the fleeces. In fact, red nuclei from dead or apoptotic cells were found only very rarely (Figure 3A, arrows). In addition, no significant difference in fleece cell survival was evidenced among samples (Figure 3B, *p* > 0.05). 

**Figure 4:**
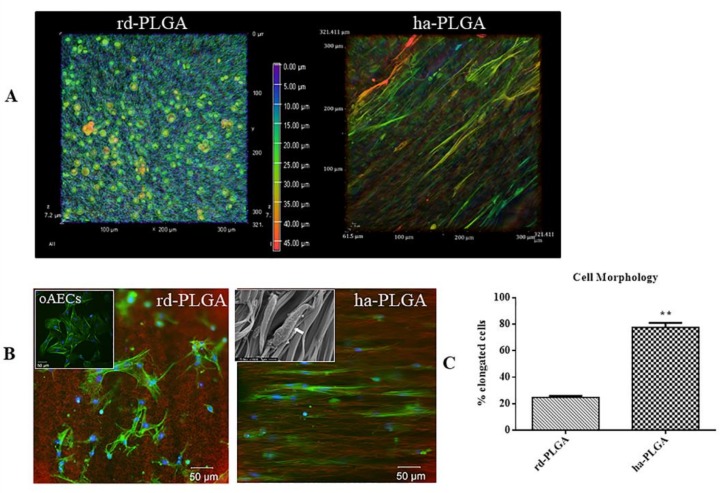
oAEC distribution and morphology on ha-PLGA and rd-PLGA fleeces. (**A**) Representative XY confocal images of oAEC distribution within rd-PLGA and ha-PLGA fleeces after 48 h culture. The depth-coded MaxIP option was used to assess cell penetration within the fleeces by defining the gradient color related to the direction of the cells within the fleece. The gradient scale shows in purple the most superficial surface of the fleece, whereas in red the bottom surface. It is evident that oAECs are optimally distributed within the fleeces, especially in ha-PLGA where there are some blue cells. (**B**) Representative images of oAEC morphology evidenced with phalloidin stain (green fluorescence) and nuclei counterstained with DAPI (blue fluorescence), where it is evident that, in rd-PLGA fleeces, cells retain their typical cobblestone morphology, whereas they acquire an elongated tenocyte-like morphology after 48 h culture on ha-PLGA fleeces. The inset in the ha-PLGA image shows an example of an SEM microphotograph where the fusiform morphology of the cells attached on the aligned fibers is evident (white arrow). Fleeces captured from bright field exposition were inverted and combined in the image as red channel. Scale bar = 50 µm. In the insert an example of oAEC morphology at time 0 cultured on a Petri dish. Scale bar = 50 µm. (**C**) Quantification of the percentage of the elongated oAECs after 48 h seeding on rd-PLGA and ha-PLGA fleeces ** Statistically significant values between rd-PLGA and ha-PLAGA fleeces (*p* < 0.01). (n = 3 for each type of fleece/analysis), scaffold size: 15 mm × 7 mm).

Cells distributed uniformly on the surface and within both types of PLGA fleeces (Figure 4A). In particular, the gradient of cell penetration analysis within the examined fleeces demonstrated an optimal cell migration and integration within the fleeces, demonstrating that cells spread inside both type of fleeces, even if in ha-PLGA the cells penetrated more in depth (Figure 4A, see the depth coded MaxIP scale). 

Actin staining with phalloidin, in combination with the background information of the PLGA fleeces, shows very clearly that the cells were strongly influenced in their morphology and in the alignment of the actin fibers by the structure of the electrospun fleeces. In particular, phalloidin stain (green fluorescence) allowed us to verify that oAECs seeded on rd-PLGA fleeces (CTR) retained their typical polyhedral morphology and ovoid nuclei and presented a random distribution according to the topography of the fibers (Figure 4B). In contrast, on ha-PLGA fleeces, oAECs acquired an elongated morphology, tenocyte-like, and aligned along the parallel-distributed fibers (Figure 4B). On these samples, alignment of the cell nuclei was also apparent in DAPI staining (blue fluorescence), as they became more uniformly oriented in the direction of the ha-PLGA fibers (Figure 4B). These observations were also assessed by the SEM analysis that clearly shows cell adhesion and morphological modifications due to the cell-material interaction. Cells acquired a fibroblastic-like morphology and the reduction of their cytoplasmic extensions demonstrate that they have well adapted to the fleece and that the cells have differentiated (inset in Figure 4B), further confirming the effect of matrix guidance on cell morphology and biology. After just 48 h culture, the percentage of oAECs with an elongated morphology was significantly higher in ha-PLGA (~80%) fleeces than in rd-PLGA fleeces (*p* < 0.01; Figure 4C). 

**Figure 5:**
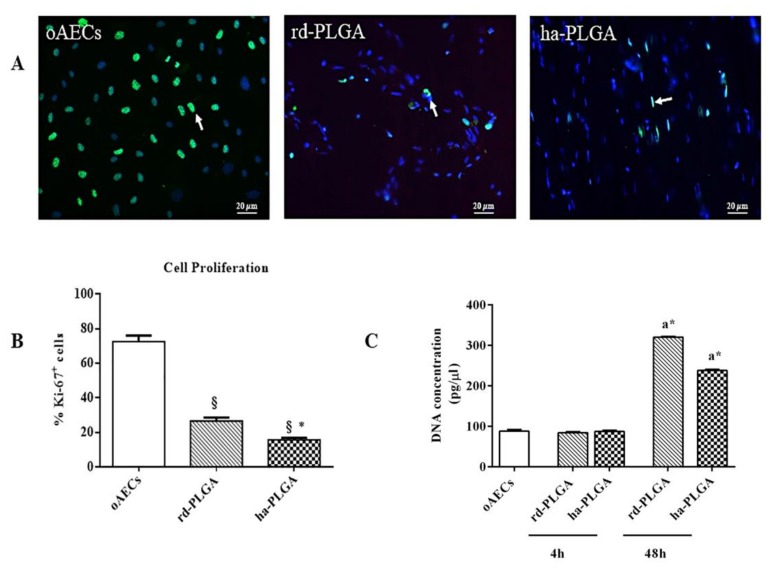
Effect of fiber alignment on oAEC proliferation. Cells were seeded on Petri dishes (oAECs) or on ha-PLGA and rd-PLGA electrospun fleeces and allowed to grow for up to 48 h. (**A**) Representative images of oAEC proliferation evidenced with a proliferation marker, Ki-67 (green fluorescence). Positivity to the antibody is localized in cell nuclei (white arrows), which were counterstained with DAPI (blue fluorescence). Scale bars = 20 µm. (**B**) Histogram representing oAEC proliferation index calculated after 48 h culture. AECs seeded onto both type of electrospun PLGA fleeces exhibit significantly lower cell proliferation activity compared to those seeded on Petri dishes; oAECs cultured on rd-PLGA fleeces are more proliferating than the ones seeded on ha-PLGA fleeces. * Statistically significant values between rd-PLGA and ha-PLAGA (*p* < 0.05). ^§^ Statistically significant values of rd-PLGA and ha-PLAGA compared to oAECs (*p* < 0.001). (**C**) DNA quantification by Qubit^®^ dsDNA HS Assay on oAECs before seeding and after 4 h on seeded fleeces to verify cell adhesion and seeding efficiency, and after 48 h of AECs seeded on scaffolds to assess DNA quantity. It is shown that there is a smaller proliferation rate in the case of ha-PLGA compared to rd-PLGA. * Statistically significant values compared to the same type of fleeces after 4 h and 48 h of cell culture (*p* < 0.05). ^a^ Statistically significant values within the different fleeces at the same time of culture (*p* < 0.05). (n = 3 for each type of sample/analysis/time point, scaffold size: 15 mm × 7 mm).

Cell proliferation was different between rd-PLGA and ha-PLGA fleeces as indicated by the expression of the Ki-67 proliferation marker (green fluorescence) in their nuclei (Figure 5A). In particular, cell proliferation index (PI) after 48 h of culture showed that oAECs on rd-PLGA were significantly more proliferating when compared to ha-PLGA (Figure 5B; *p* < 0.05), even if the cells cultured on both types of fleeces proliferated less than the oAECs standardly cultivated in a Petri dish (Figure 4B; *p* < 0.001). 

DNA quantification of 0.05 × 10^6^ oAECs before seeding (oAECs: 91.66 ± 3.33 pg/µL) was comparable to the values obtained after 4 h of cell seeding on fleeces (rd-PLGA: 86.62 ± 1.26 pg/µL; ha-PLGA: 87.54 ± 1.75 pg/µL, *p* > 0.05; Figure 5C) and to those obtained after 4 h of the cells cultured on a Petri dish (90.94 ± 1.42 pg/µL, data not shown). These results confirm a high cell seeding and adhesion efficiency on the electrospun PLGA fleeces. In addition, after 48 h, an increased DNA quantity was evident in seeded fleeces in comparison to those observed after 4 h of culture (*p* < 0.05; Figure 5C). In particular, after 48 h of culture, oAEC DNA quantity was significantly higher in rd-PLGA (320.46 ± 1.17 pg/μL) compared to ha-PLGA (238 ± 1.67 pg/μL: *p* < 0.05; Figure 5C) with a different growth rate on the cell population within the two seeded fleeces, confirming the PI results related to the influence of the different fleece fiber topography. 

### 3.3. ha-PLGA Fleeces Induce oAEC Epithelial-Mesenchymal Transition

**Figure 6:**
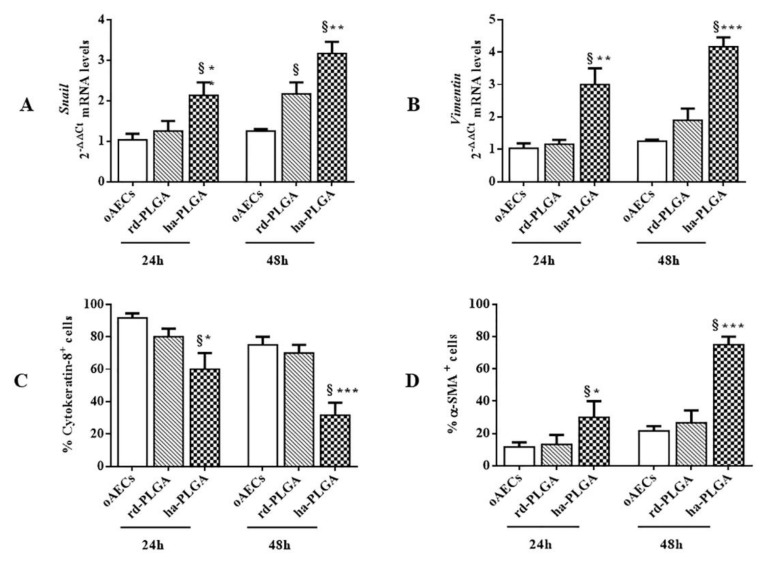
Epithelial-Mesenchymal Transition (EMT) gene and protein expression in oAECs cultured on Petri dishes or onto electrospun rd-PLGA and ha-PLGA fleeces after 24 h and 48 h. Results show that electrospun ha-PLGA fleeces were able to modulate oAEC phenotype by accelerating the EMT. Quantitative RT-PCR data show a significantly higher expression of (**A**) *Snail* and (**B**) *Vimentin* mRNA expression when oAECs are seeded onto ha-PLGA compared to the other groups. Immunohistochemistry (IHC) analysis was used to evaluate EMT by quantifying the percentage of positive cells for (**C**) Cytokeratin-8 and (**D**) α-SMA protein expression * Statistically significant between ha-PLGA and rd-PLGA (*p* < 0.05). ** Statistically significant between ha-PLGA and rd-PLGA (*p* < 0.01). *** Statistically significant between ha-PLGA and rd-PLGA (*p* < 0.001). ^§^ Statistically significant between ha-PLGA and oAECs (*p* < 0.001). (n = 3 for each type of sample/analysis/time point, scaffold size: 15 mm × 7 mm).

Since oAECs lost their cobblestone morphology and almost all became elongated in 48 h, it was verified if an EMT process occurred in these stem cells by analyzing their genotypic and phenotypic profile. In particular, the expression of the mRNAs of the EMT-transcription factor *Snail* and of the mesenchymal marker *Vimentin* were analyzed by RT-qPCR. As shown in Figure 6 *Snail* and *Vimentin* mRNA expressions were analyzed at 24 and 48 h of oAEC culture on both type of fleeces and on a Petri dish, as a control. *Snail* gene expression was significantly upregulated early at 24 h in oAEC-ha-PLGA with respect to oAEC-rd-PLGA and oAECs (Figure 6A; *p* < 0.01 and *p* < 0.001, respectively). *Snail* significantly increased further at 48 h compared to cells cultured alone or onto rd-PLGA bio-hybrid fleeces (Figure 6A; *p* < 0.05). In turn, *Vimentin* mRNA expression significantly and progressively increased in oAEC-ha-PLGA compared to rd-PLGA bio-hybrid fleeces (Figure 6B; *p* < 0.01 at 24 h and; *p* < 0.001 at 48 h) and with respect to oAECs at both time points (*p* < 0.001). 

Ovine AEC EMT gene transition was confirmed also by analyzing the phenotype. In detail, Cytokeratin-8, a typical epithelial marker, and a-SMA, a typical mesenchymal marker, protein expressions were evaluated by immunostaining in oAECs engineered on PLGA fleeces after 24 and 48 h of culture and the number of positive cells to these two markers was quantified. AECs started to have a significant decrease in Cytokeratin-8 expression when seeded onto ha-PLGA fleeces with respect to oAECs cultured alone or seeded on rd-PLGA fleeces after 48 h culture (Figure 6C; *p* < 0.05), whereas no changes were observed in the other experimental conditions. At the same time, a progressive and significant increase in the EMT protein marker expression of α-SMA in oAECs cultured on ha-PLGA occurred, with respect to those observed on cells cultured on rd-PLGA or alone on a Petri dish (Figure 6D; *p* < 0.05).

### 3.4. ha-PLGA Fleece Possesses Teno-Inductive Properties on oAECs 

**Figure 7:**
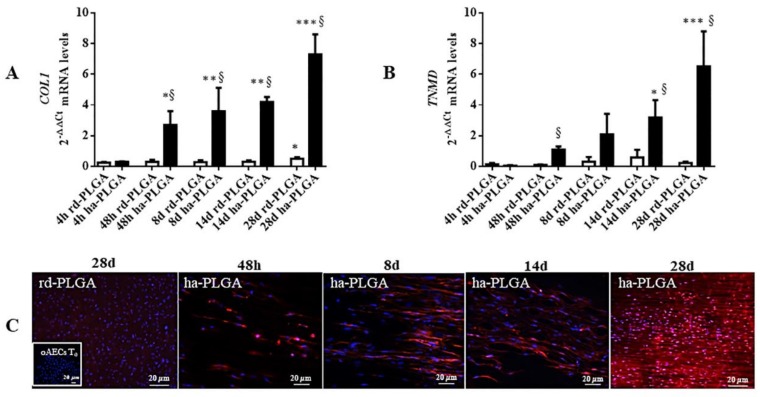
Teno-inductive properties of ha-PLGA electrospun fleeces on AECs. *COL1* and *TNMD* gene expression, and COL1 protein immunopositivity were evaluated at different culture points. Quantitative RT-PCR data show a significantly higher expression in oAECs seeded onto ha-PLGA fleeces of (**A**) *COL1* mRNA expression starting from 48 h culture, whereas (**B**) *TNMD* mRNA expression started to be upregulated after 14 days culture. *COL1* and *TNMD* gene expression were always lowly expressed in rd-PLGA at all culture time points. * *p* < 0.01 different values in the same fleece type. ** *p* < 0.001 different values in the same fleece type. *** *p* < 0.0001 different values in the same fleece type. ^§^ *p* < 0.01 different values between different fleeces in each time. (**C**) IHC analysis revealed COL1 protein expression (red fluorescence) in oAECs when seeded onto ha-PLGA and rd-PLGA; whereas freshly isolated cells do not express the protein (inset). Nuclei were counterstained with DAPI (blue fluorescence). Images show that cells seeded onto rd-PLGA never express COL1, whereas it is expressed in AECs cultured on ha-PLGA fleeces already after 48 h culture. Up to 14-day positivity to COL1 was evident in the cytoplasm, whereas at day 28 positivity to the protein was localized also within the fleece. Scale bars = 20 μm. (n = 3 for each type of sample/analysis/time point, scaffold size: 15 mm × 7 mm).

PLGA fleece teno-inductive properties were tested on the seeded oAECs. Cells were cultured, without adding any growth factor, on ha-PLGA and rd-PLGA (CTR) for 4, 48 h, and 8, 14, 28 days. The tenogenic differentiation was studied by analyzing mature tendon-specific markers *COL1* and *TNMD* gene expression and COL1 protein expression (Figure 7). RT-qPCR results showed that oAECs exhibited significantly higher levels of *COL1* gene expression when seeded onto the electrospun ha-PLGA fleeces compared to the rd-PLGA fleeces, starting from 48 h culture with the highest expression values detected after 28 days of culture (Figure 7A; *p* < 0.05). In rd-PLGA, after 28 days of culture, there was a *COL1* upregulation in the oAECs with respect to the other culture time points (Figure 7A; *p* < 0.05). In contrast, *TNMD* gene expression started to be significantly upregulated only starting from 14 days of culture (Figure 6B; *p* < 0.05), but it was significantly higher in respect to rd-PLGA after 48 h of culture. AECs seeded on these fleeces always maintained low *TNMD* expression levels (Figure 7B).

Importantly, while oAECs normally do not express COL1 (Figure 7C inset), the fiber alignment in the electrospun ha-PLGA fleeces induced COL1 production in oAEC cytoplasm already after 48 h (Figure 7C). In addition, in ha-PLGA fleeces, COL1 protein was also secreted extracellularly, guiding protein formation along the arrangement direction of fibers (Figure 7C). On the contrary, in rd-PLGA no COL1 protein expression could be found even after 28 day of culture (Figure 7C). Together, these results demonstrated that ha-PLGA fleeces provided a more appropriate microenvironment to promote tenogenic differentiation of oAECs.

### 3.5. ha-PLGA-oAEC Bio-Hybrid Fleece Co-Culture with Fetal Tendons Accelerates Tenogenic Differentiation

In order to verify if it was possible to accelerate oAEC tenogenic differentiation when seeded on ha-PLGA fleeces, fetal tendon explants were allowed to co-culture in a transwell system with the oAECs seeded on both type of fleeces for 14 and 28 days. 

**Figure 8:**
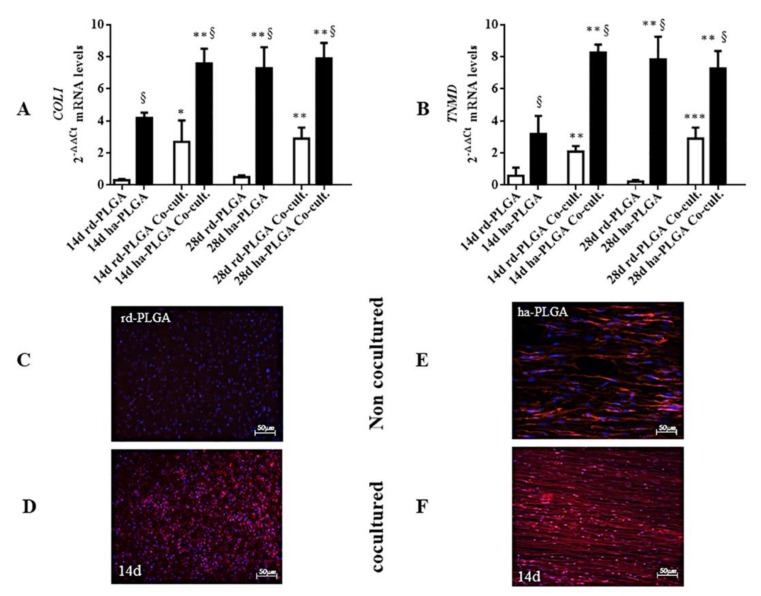
oAEC tenogenic differentiation analysis in electrospun PLGA fleeces after using the normal or co-culture system with fetal tendon explants after 14 and 28 days. Quantitative RT-PCR for (**A**) *COL1* and (**B**) *TNMD* mRNA expression showing that oAECs seeded onto ha-PLGA already at 14-day co-culture highly produce the two tenogenic markers with values that are comparable to the corresponding 28-day normal condition cultured cells. Co-cultured oAEC-rd-PLGA fleeces started to express *COL1* and *TNMD* from day 14. * *p* < 0.01 different values in the same fleece type. ** *p* < 0.001 different values in the same fleece type. *** *p* < 0.0001 different values in the same fleece type. ^§^ *p* < 0.01 different values between different fleeces in each time culture. (**C**,**D**) IHC shows that in rd-PLGA fleeces, after 14 days culture, oAECs start to faintly express COL1 in their cytoplasm in co-culturing conditions. Scale bars = 50 μm. (**E**,**F**) At day 14 positivity to COL1 was evident in the cytoplasm in the normal cultural condition, whereas in the co-culturing condition oAECs secreted the protein also in the fleece. Scale bars = 50 μm. (n = 3 for each type of sample/analysis/time point, scaffold size: 15 mm × 7 mm).

The gene expression of tenogenic differentiation markers, *COL1* and *TNMD*, in ha-PLGA and rd-PLGA was evaluated by RT-qPCR. The analysis showed that the co-cultured ha-PLGA-oAEC bio-hybrid significantly upregulated *COL1* and *TNMD* gene expression already at day 14, compared to the corresponding fleeces normally cultured at the same time point (Figure 8A,B; *p* < 0.01), showing that the tendon-related genes were significantly upregulated in ha-PLGA-oAECs in half the time with the co-culture system. The co-cultured or normally cultured rd-PLGA-oAEC bio-hybrids alone always showed the tendon-related markers significantly lower to the ha-PLGA ones (Figure 8A,B; *p* < 0.05). Although, co-cultured rd-PLGA-oAEC bio-hybrids showed an upregulation of *COL1* and *TNMD* gene expression especially at day 28 (Figure 8A,B; *p* < 0.05).

Immunofluorescent staining showed that the protein expression of COL1 was nearly non-detectable after 14 days of culture in oAECs seeded on rd-PLGA fleeces (Figure 8C). On the contrary, a minimal COL1 expression was detected in the co-cultured rd-PLGA-oAEC bio-hybrid (Figure 8D). Protein expression was detected in both normally and co-cultured ha-PLGA-oAEC bio-hybrid groups already after a 14-day culture (Figure 8E,F), but in the co-cultured one the immunostaining resembled the ECM architecture of native tendon tissues already at day 14 of culture (Figure 8F), whereas in the normally cultured ha-PLGA-oAEC bio-hybrid after 28-day culture (Figure 7C), showing also with this the technique an anticipated process of cell differentiation.

## 4. Discussion

In the present study, PLGA tendon biomimetic fleeces were fabricated via electrospinning with a high reproducible quality. In particular, the produced electrospun ha-PLGA fleeces were characterized by highly aligned fiber topography and mechanical properties that mimic the native tendon ECM. Furthermore, the aligned fleece induced oAEC tenogenic differentiation through an EMT-mediated pathway. Thus, this research demonstrates the correlation between fiber alignment of the fleece and the differentiation of oAECs towards a mesenchymal lineage such as the tenogenic one, suggesting that the alignment of the fibers can potentially stimulate and sustain tendon regeneration.

Electrospinning, the fleece fabrication technology used in this research, is relatively economical, controllable, reproducible, and suitable and has encouraged researchers to use it in tissue engineering, especially for in vivo studies [46,47]. Even if natural substances such as collagen and fibrin [48] are more biocompatible, many synthetic polymers have been used in tendon tissue engineering such as PCL [14,49], PLLA [47,50], chitosan [51], a mixture of polymer PLLA/PCL [52], PCL/Gel [53,54], and PLGA, which has been widely used in tendon tissue engineering [16,40,55,56,57]. This material has been chosen to fabricate the fleece used in this work since it is FDA-approved and provides sufficient control of degradation [58,59] combined with a sufficient mechanical strength that fosters its application for tissue remodeling and regeneration [60,61]. Moreover, in a previous study it has been demonstrated that PLGA is highly biocompatible for oAECs, the cells chosen to test the fabricated fleeces, and that the chemistry of the fleece influences their penetration and distribution within the construct, efficiently sustaining and stimulating cell adhesion, viability, and proliferation [62].

To generate engineered tendon grafts, the bio-mimicking of the ECM topography and its mechanical properties are of significant importance since in vivo mechanical forces influence tendon activity [2,19]. Native tendons are characterized by a hierarchical structure constituted of parallel bundles of collagen fibers oriented along the longitudinal axis of the tissue, which are needed to ensure connective flexibility between muscles and bones and transmit tensile forces. In this work, fiber diameter size was adjusted to the micrometer range, in contrary to most published papers in which fibers were prepared in the nanometer range [16,52,54,55], since tenocytes reside on the surface of collagen fibers. The fabricated ha-PLGA fleeces with their highly aligned fibers had a structure and organization similar to the native tendon ECM—highly-aligned fiber bundles of collagen [63]. It was clearly seen that the orientation of the fibers is modulated by varying the rotational speed leading to more uniform fibers when higher rotational speed is applied. These results are consistent with previous works that studied the effect of rotational speed on fiber alignment and orientation [50,64]. Moreover, the ha-PLGA fleeces, different from the rd-PLGA ones used as control, closely mimic the biomechanical properties of a native tendon, as in fact, especially the stress values were comparable to those of the human tissues (i.e., human Achilles, rotator, and patellar tendons) [2]. In detail, the stress values of the produced ha-PLGA fleeces (26.02 ± 1.75 MPa) closely resembled those published for human Achilles tendons (28–86 MPa) [2,65], human patellar tendons (5–65 MPa) [2,66], human rotator tendons (14–45 MPa), and human Achilles grafts (16 MPa) [2]. These obtained data are in accordance with those published previously and confirm that the mechanical properties depend on the fiber orientation and possess higher mechanical strength when the fibers are aligned, compared to the randomly distributed ones [16,56,67]. Thus, these results demonstrate that fleece fibers produced by electrospinning not only were organized in a parallel manner, creating a defined orientation, but also mimicked tendon sufficient mechanical properties.

Since the PLGA fleeces are used for biological applications, the sterilization procedure is of critical concern. In this research, it has been demonstrated that the used sterilization procedure of PLGA fleeces in EtOH did not alter PLGA chemical composition. The chemistry of the fleece was confirmed not to impair oAEC viability. Moreover, the topography of the fibers influenced not only the morphology of the cells but also their state of differentiation. Considering that these stem cells are epithelial, showing a typical polyhedral morphology, it has been demonstrated that highly aligned PLGA fleeces can modify their genotypic and phenotypic profiles through an EMT mechanism, and can induce their tenogenic differentiation by producing a tendon-like ECM containing oriented COL1 fibers. Thus, ha-PLGA fleeces were also teno-inductive for oAECs as demonstrated also by analyzing their tendon-specific gene expression profile. 

Indeed, after only 48 h culture, oAECs were influenced by the topology of the fleece and acquired a spindle tenocyte-like shape, whereas in randomly oriented fiber rd-PLGA fleeces, used as control, the cells retained their native morphology. As supported by prior studies, the findings herein presented confirm that the aligned direction of fibers leads to a cell alignment and organization along the fiber arrangement [14,47,51,68,69,70]. 

Additionally, oAECs cultivated on ha-PLGA fibers showed a statistically reduced PI compared to rd-PLGA. The reduced PI and DNA quantification observed could be a consequence of the oAEC pre-commitment towards the tenogenic lineage when cultured on ha-PLGA fleeces. It is conceivable that the rapid morphological elongation of oAECs on the aligned fibers caused an earlier EMT response followed by a teno-inductive differentiation signal that stopped their proliferation. Physiologically, both the hAEC and oAEC in vitro expansions induce a spontaneous loss of their epithelial phenotype with a consequent EMT, but this process normally occurs after 3 passages (i.e., about 21–28 days) [36]. EMT is a complex biological process pivotal in development, wound healing, and stem cell differentiation, but in a pathological state it is involved also in supporting organ fibrosis and cancer progression [71]. EMT is not only involved in physiological and pathological functions, but recently it has been demonstrated that in in vitro conditions might be responsible for varying cell functions [36,72,73]. A family of transcription factors (EMT-TFs) such as *Snail*, *Twist,* and *ZEB* regulates EMT. These EMT-TFs are responsible for the downregulation of epithelial genes and proteins (E-Cadherin, Cytokeratin-8) and for up-expression of the mesenchymal ones (*Vimentin*, α-SMA) [74,75,76]. Indeed, the precocious mesenchymal genotypic and phenotypic changes of oAECs due to their culture on ha-PLGA fleeces were further established by the upregulation of EMT-related genes (*Snail* and *Vimentin*) and by the α-SMA (mesenchymal marker) protein expression, with a significant decrease of the Cytokeratin-8 (epithelial marker) protein expression compared to those detected on engineered rd-PLGA control fleeces. This interesting result demonstrates that an aligned topography exercises an instructive function on cells by strongly influencing their biology. The obtained results are in line with the observation on the epithelial MCF-7 cells that were grown on a collagen fibrillar network underwent the EMT by strongly increasing their mesenchymal markers (vimentin, fibronectin), whereas their epithelial marker (E-cadherin) was decreased and also their cell morphology was modified [77]. Even if a direct relation between EMT and tenogenic lineage differentiation has not been reported yet, it has been demonstrated using a tendon injury model that shows genes that play a role in EMT processes such as *Snail1*, *Slug*, *Goosecoid,* and *Twist1* are coordinately regulated during the tendon regeneration [78,79]. Moreover, it has also been demonstrated in vitro that oAECs undergo EMT when co-cultured with tendon explants [33], and recently demonstrated that an in vivo stepwise hAEC trans-differentiation occurs after their xenotransplantation in an ovine tendon injury model. In particular, microarray analysis showed that among the 49 upregulated human transcripts hAECs expressed following transplantation were both the epithelial-mesenchymal transition genes (e.g., *KDM6B*) and connective tissue genes such as *COL1*, *COL11A1*, and *COL21A1* [80].

Ovine AEC transition from an epithelial to a mesenchymal phenotype was brought further as the seeded cells on ha-PLGA fleeces differentiated into tenocyte-like cells, as shown by a significant tendon-specific gene marker upregulation (*TNMD, COL1*) and COL1 protein expression in their cytoplasm. In particular in ha-PLGA bio-hybrids, oAECs started to express *COL1* and *TNMD* mature tendon-specific genes [22], as well as the COL1 protein already after 48 h culture without adding any tenogenic differentiation media. Thus, oAECs were subjected to an accelerated EMT and tenogenic differentiation due to the topology of the fibers constituting the ha-PLGA fleeces. These results are very encouraging, since in other publications where mesenchymal stem cells were used on aligned fibers, type 1 collagen protein started to be expressed after 3 [16,56] and 7 days [55] of culture, while AECs used in this work that usually do not express this protein were able to express *COL1* mRNA and protein after just 48 h of culture. In addition, other papers showed also that cells on aligned scaffolds started to express tendon-related genes after 3 [54], 7 [14,55], 14 [51], and 21 days [50] of culture, whereas in this research *TNMD* was upregulated just after a 48-h culture with respect to the rd-PLGA fleece, with a further significant upregulation after 14 days culture. Moreover, ovine AEC teno-differentiation increased during the long-time cultures (8, 14, and 28 days), but more importantly, after 28 days culture COL1 was synthesized and deposited extracellularly within the ha-PLGA fleece. 

This COL1 production and deposition was further accelerated when bio-hybrid oAEC-ha-PLGA fleeces were co-cultured with fetal tendon explants. Indeed, in this type of tissue bioactive molecules that are necessary to promote tendon tissue formation and maturation are found. In particular, it is characterized by high expression of several tendon-specific genes, including *COL1, COLIII, Scleraxis B, TNMD, Thrombospondin 4,* and *Osteocalcin*, and growth factors such as *TGF-ß1* (involved in collagen synthesis) and *VEGF* (involved in the angiogenesis), essential for the homeostasis of the tissue, to then undergo a dramatic reduction in adult tissues [81]. Recently it has been demonstrated that the co-culture system enhances specific tissue formation facilitated by the cell–paracrine interaction, and this technique has an enormous prospective in tissue engineering applications [33,82]. In particular, Barboni et al. demonstrated that the co-culture system improved oAEC tenogenic differentiation efficiency thanks to the fetal tendon explants that release tenogenic-soluble factors [33]. It is possible to exploit the release of these factors also in xeno-co-culture conditions, as demonstrated by Muttini et al., by using equine tendon explants with oAECs allowing their tenogenic differentiation [83]. This allows us to hypothesize, in order to create engineered tendon constructs for human tendon regeneration, the possibility of using a xeno-co-culture system with hAECs and ovine fetal tendon explants. In the present study, fetal tendon explants were co-cultured with oAECs seeded on the PLGA electrospun fleeces. This co-culture system accelerated oAEC tenogenic differentiation by halving the time of culture. Indeed, by co-culturing fetal tendons with the oAEC-ha-PLGA fleeces, cells markedly upregulated *COL1* and *TNMD* gene expression already at day 14 showing similar expression values and COL1 deposition of oAECs cultivated under normal conditions after 28 days. In contrast, oAECs cultured on rd-PLGA never expressed tendon-specific markers, whereas they were produced at very low concentrations and expression in oAEC-rd-PLGA bio-hybrids co-cultured with the tendon fetal explants, probably due to the influence exerted by the paracrine-soluble factors released by the tissue on the cells. The indirect co-culture of oAECs and the fetal tendon explants has promoted and accelerated the tenogenic differentiation of oAECs and, thus it could speed up tendon-specific tissue regeneration. In fact, it is envisioned that the strategy to combine the developed ha-PLGA fleeces with concurrent stimulation of oAECs by co-culturing them with tendon explants could be effectively utilized for tissue engineering to innovatively regenerate tendon tissue constructs. 

Overall, the obtained results indicate that fleece topography plays a crucial role in cell function and in this particular case in cell trans-differentiation towards the tenogenic lineage. Even if it is commonly regarded that the cell fate is controlled by soluble factors, matrix topography gives equally important instructions in controlling oAEC gene expression and its fate. These results suggest that by bio-mimicking tendon ECM, the attachment of oAECs to the aligned fibers promotes their teno-differentiation. Thus, this study also offers a valuable approach to stimulate tendon differentiation and demonstrates that oAECs are a smart model for cell–matrix interface studies and tendon regeneration. These investigations on the mechanism of oAEC commitment induced by the fiber topography emphasize the crucial role of the interaction between the cell and the matrix and the mechano-chemical signals finally regulating stem cell fate. Future studies are still required to validate in vivo these tendon-engineered bio-hybrids. The fabricated ha-PLGA fleece has functional and biocompatible properties that could lead to the development of engineered constructs able to promote tissue formation by organizing and aligning cells that facilitate required interactions between cells. The guidance of tenocyte-like cells by fibers and the collagen matrix orientation in vivo may support the achievement of the regenerated tendon to its native functions.

## 5. Conclusions

In the present study, novel, differently structured PLGA constructs that mimic the native tendon ECM in its architecture and biomechanics were developed using the electrospinning technology. This fleece showed the required mechanical properties similar to human tendons. The sterilization process did not alter the chemical composition of PLGA. In addition, it possessed a microstructure which was favorable for cell alignment, proliferation, penetration, and the expression of EMT and tendon-related markers, demonstrating its teno-inductive properties. More importantly, using oAECs that have an epithelial genotype and phenotype in origin as a stem cell model allowed verification of the precocious EMT mechanisms occurring to oAECs when cultured on the PLGA constructs possessing highly aligned fibers. Moreover, the cultured oAEC-ha-PLGA fleeces induced a significant cellular tenogenic differentiation by enhancing their collagen expression, and by upregulating the expression of tendon-related genes. Thus, this study demonstrates that an instructive microenvironment for oAECs is provided by the aligned electrospun fibers, since they are trans-differentiated into mesenchymal cells of the teno-lineage. This research contributes to understanding the biological activity of oAECs on highly aligned fibrous PLGA fleeces and could be useful in developing effective engineered tendons consisting of ideal stem cells and innovative fleeces that can arrange in a parallel manner cell population, facilitating their tenogenic differentiation. Moreover, it has also been demonstrated that the ha-PLGA constructs could serve to enhance by properly combining cell co-culture and by promising tissue-engineered fleeces with the tenogenic differentiation for tendon regeneration. In particular, the favorable interaction between oAECs, which have a considerable tenogenic inclination [32,33], and ha-PLGA fleeces allows us to consider this bio-hybrid construct an ideal combination for tendon tissue engineering.

## Figures and Tables

**Table 1:** Details of primary and secondary antibodies used for Immunohistochemistry (IHC).

Primary Antibody	Dilution	Supplier	Secondary Antibody	Dilution	Supplier
**Ki-67**	1:50	Dako Cytomation, Glostrup, Denmark	Anti-Mouse Alexa Fluor 488	1:100	Invitrogen, Paisley, UK
**Phalloidin–FITC**	1:40	Sigma-Aldrich, St. Louis, MO, USA			
**Cytokeratin-8**	1:200	Abcam, Cambridge, UK	Anti-Mouse CY3	1:500	Sigma-Aldrich, St. Louis, MO, USA
**α** **-SMA**	1:500	Abcam, Cambridge, UK	Anti-Mouse FITC	1:100	Sigma-Aldrich, St. Louis, MO, USA
**COL1**	1:100	EMD Millipore Corporation, Temecula, USA	Anti-Mouse CY3	1:500	Sigma-Aldrich, St. Louis, MO, USA

**Table 2:** Details on primers and probe sequences.

Gene	Forward Primer (5′ to 3′)	Reverse Primer (5′ to 3′)
*Vimentin*	GACCAGCTCACCAACGACA	CTCCTCCTGCAACTTCTCCC
*Snail 1*	GTCGTGGGTGGAGAGCTTTG	TGCTGGAAAGTGAGCTCTGG
*GAPDH*	CCTGCACCACCAACTGCTTG	TTGAGCTCAGGGATGACCTTG
*COL1*	AGAAGAAGACATCCCACCAGTCA	GGCAGGGCACGGGTTT
	Probe	AGAACGGCCTCAGGTACCATGACCG
*TNMD*	AGCACTTCTGGCCTGAGACAC	CAGTGCCATTTCCACTTCTGAAT
	Probe	AGATTTACATGGAAATTGATCCCATTACCAGAACTG
*GAPDH*	GGCGTGAACCACGAGAAGTATAA	CCCTCCACGATGCCAAAGT
	Probe	ATACCCTCAAGATTGTCAGCAATGCCTCCT
